# Baltic amber teething necklaces: could succinic acid leaching from beads provide anti-inflammatory effects?

**DOI:** 10.1186/s12906-019-2574-9

**Published:** 2019-07-05

**Authors:** Michael D. Nissen, Esther T. L. Lau, Peter J. Cabot, Kathryn J. Steadman

**Affiliations:** 1 University of Queensland, School of Pharmacy, 20 Cornwall St, Woolloongabba, QLD 4102 Australia; 2 Queensland University of Technology, School of Clinical Sciences, 2 George St, Brisbane, QLD 4000 Australia

**Keywords:** Amber, Teething, Infant care, Inflammation, Analgesia, Succinic acid, NSAIDs

## Abstract

**Background:**

Baltic amber teething necklaces have been popularized as a safe and natural alternative to conventional or pharmacological medicines for the management of teething pain. However, claims made by retailers regarding the efficacy and mechanism of action of these necklaces lack scientific or clinical basis. The claim most closely resembling science is the assertion that succinic acid will leach out of the beads and through the skin of the wearer and carry out anti-inflammatory and analgesic effects. The objective of the current research is to scientifically assess this claim.

**Methods:**

Beads from necklaces were powdered for identification by infrared spectroscopy, and dissolved in sulfuric acid for quantification of succinic acid using HPLC. Succinic acid release from beads was assessed by long-term submersion of amber beads (separated according to light, medium and dark brown colour) in solvents relevant to human skin conditions. The potential for succinic acid to have anti-inflammatory effects was assessed by measuring the release of inflammatory cytokines IL-1α, IL-1β, IL-8 and TNFα, and the inflammatory messenger PGE2, from THP-1 human macrophages after treatment with succinic acid and LPS.

**Results:**

Amber teething necklaces were positively identified as Baltic amber, by comparison of the beads’ infrared spectrum to the literature, and by their succinic acid content (1.5 mg per bead; 1.44% w/w). However, whole amber beads submerged in octanol or pH 5.5 phosphate buffered saline did not release any measurable succinic acid, except for the light-coloured beads in octanol which broke into tiny fragments. Additionally, treatment of macrophages with succinic acid did not reduce the release of any inflammatory cytokines measured, and displayed toxicity to the cells at high concentrations.

**Conclusions:**

While amber teething necklaces are genuine Baltic amber, we have found no evidence to suggest that the purported active ingredient succinic acid could be released from the beads into human skin. Additionally, we found no evidence to suggest that succinic acid has anti-inflammatory properties.

## Background

Over the last decade, Baltic amber teething necklaces have been promoted by various organizations and individuals as an alternative to conventional medicines for relief from symptoms of teething in infants. Claims surrounding the efficacy and mechanism-of-action by these beads range from the mystical, such as “bio-transmitter”, to the pseudoscientific, such as “electromagnetic” [1–4].

However, some retailers claim that the amber beads release succinic acid, which is then absorbed through the skin, to provide analgesia and anti-inflammatory effects [2–5]. This provides a somewhat scientific basis for teething relief, given several inflammatory cytokines, such as interleukin (IL)-1β, IL-10 and tumour necrosis factor (TNF)-α, play a role in the pain and discomfort associated with the teething process [6]. Conventionally, this inflammation is inhibited by using non-steroidal anti-inflammatory drugs (NSAIDs) like ibuprofen in the form of oral liquids, or as topical gels containing salicylate applied directly to the gums of teething infants. A common mechanism by which these drugs work is by reduction of prostaglandin synthesis via inhibition of cyclooxygenase (COX) [7]. Indeed, Baltic amber (mineralogical name ‘succinite’), is a fossil resin that characteristically, and in contrast to most other fossil resins, contains succinic acid. However, succinic acid is primarily present conjugated via ester bonds to the hydroxyl group of other compounds [8] and through cross-linking these compounds it plays a structural role in Baltic amber [9] so it is difficult to conceptualise how succinic acid could be released from amber bead jewellery.

In 2011, the Australian Therapeutic Goods Administration (TGA) issued warnings to two amber teething necklace retailers, Eco-Child and Nature’s Child, ordering these companies to substantiate their advertised claims that amber teething necklaces provide analgesic and anti-inflammatory properties. When neither of these retailers could do so, the TGA then ordered the advertisements to be retracted in 2013, under the determination they were misleading to the public [10, 11]. The strangulation hazard of teething necklaces has also been highlighted in the past, with both the Australian Consumer and Competition Commission (ACCC) and the Queensland Government issuing product safety warnings regarding the risk of choking or strangulation posed by teething necklaces [12, 13]. Along these lines, a survey of parents who used teething necklaces as a remedy was carried out in France in 2012, with researchers finding that only 8% of teething necklaces were sold with information regarding the risks associated with their use, but also that the parental “irrational fear of seeing their child suffer [symptoms of teething] surpassed their fear of the risk of strangulation” posed by the necklaces [14]. Indeed, one such incident of infant strangulation by an amber teething necklace has been reported as a case study [15].

The lack of scientific evidence either demonstrating or refuting the claims of retailers therefore leads to the objectives of the current research, which is to investigate the claims that 1) Amber teething necklaces are made from Baltic amber beads, 2) Baltic amber teething necklaces contain succinic acid, 3) Succinic acid can be released from Baltic amber beads at body temperature, and 4) Succinic acid has anti-inflammatory activity.

## Methods

### Materials

Baltic amber teething necklaces (33 cm Prestige Necklace, Little Smiles Genuine Baltic Amber Beads, Narangba, AUS) were purchased from a baby and toddler supplies store in Brisbane, Queensland. Individual beads were stratified by colour into light, medium and dark categories, based on some claims that lighter coloured beads contained more of the active ingredient, succinic acid [16]. Average bead weight was obtained by individually weighing 85 beads (24 light, 35 medium, 26 dark). Succinic acid, paracetamol, ibuprofen, hydrocortisone and phorbol myristate acetate (PMA) were purchased from Sigma Aldrich (St Louis, USA). Lipopolysaccharide (LPS) from *E. coli* was purchased from Enzo Life Sciences (Farmingdale, USA).

### Infrared spectroscopy

Beads contained in a sealed plastic bag were crushed into a fine powder using a hammer. The powder was analysed using an infrared spectrometer (Shimadzu IRPrestige-21 FTIR) using 20 scans for transmittance at the highest resolution setting, and transmittance was analysed using IRsolution 1.3 software (Shimadzu). The spectra were compared with published data for Baltic amber [17] after adaptation using the WebPlotDigitizer v3.21 tool.

### Succinic acid content determination

The melting point of powdered beads was 360 °C, as determined using Crown Scientific Melting Point Apparatus. As succinic acid melts at 185 °C and boils at 235 °C, an alternative method was needed in order to separate succinic acid from the amber without having to melt the beads using extremely high temperatures. Therefore, a method was developed to dissolve the beads. The beads were immersed in a range of solvents, including octanol, methanol, hydrochloric acid, or hexane. Sulfuric acid was found to affect the beads, so six beads (comprised of two beads from each colour group) were dissolved in 20 mL of concentrated H_2_SO_4_ for 16 h. 5 mL aliquots (3 replicates) of the bead-acid solutions were then neutralized using 11 mL of 18 M NaOH for analysis by HPLC for succinic acid content. The peak area of the dissolved beads was compared with a standard curve of succinic acid prepared in sulfuric acid and neutralised in the same way.

### Succinic acid release assay

To investigate if the beads would release succinic acid under various conditions, 22 beads of each colour were submerged in 10 mL of either phosphate buffered saline (PBS) at pH 5.5 (approximate pH of human skin) or octanol (organic phase to mimic hydrophobic conditions of human skin layers). Beads were left in these solutions for up to 24 weeks at 37 °C, and samples of supernatant were collected after 4, 8, 12, 16, 20 and 24 weeks and analysed by HPLC for succinic acid content. Succinic acid was quantified using a standard curve of succinic acid dissolved in PBS (0.005–10 mg/mL) or octanol (0.005–0.1 mg/mL).

### HPLC

The samples were filtered using 0.45 μm PVDF membrane filters. Following filtration, samples (50 μL) were injected on a Shimadzu HPLC using a 250 × 4.6 mm Vydac Denali C18 column (Grace Davison Discovery Sciences, Illinois, US) fitted with a guard column. The mobile phase was isocratic sulphate buffer comprised of 1 mM sulfuric acid and 8 mM sodium sulfate with pH 2.7, run at 1 mL/min for 20 min. A diode array detector (Shimadzu Prominence SPD-M20A) recorded absorbance across the range 200–800 nm with concentrations calculated using the area under the curve at 210 nm. This method for quantification was validated for linearity, specificity, sensitivity (limit of detection (LOD) and quantitation (LOQ)), and inter-day repeatability in accordance to the International Conference on Harmonization guidelines [18]. A 5-point calibration curve was constructed with standard solutions in the range of 0.005–10 mg/mL in PBS (pH 5.5). This was prepared in triplicate with the linearity assessed using linear regression analysis by least squares. Specificity was examined by contrasting the wavelength of the samples and standards across a range of 200–400 nm to determine whether bead excipients or other contributing factors affected the maximum absorbance wavelength. Sensitivity was determined by calculating the LOD = 3.3σ/S, and the LOQ = 10σ/S, with σ = standard deviation of the response and S = slope of the calibration curve. Intermediate precision (inter-day repeatability) was performed by calculating the % of relative standard deviation (%RSD) of 7 measurements for standard solutions at 0.1 mg/mL in PBS or octanol conducted over a period of 6 months.

### Assessment of anti-inflammatory activity

The potential anti-inflammatory properties of succinic acid were tested using the THP-1 human monocyte cell line (ATCC No TIB-202) [19]. THP-1 cells (2 × 10^5^) were differentiated into macrophages by stimulation with 50 nM PMA [19], before being treated with various control drugs (paracetamol, ibuprofen or hydrocortisone) or with succinic acid. Paracetamol and ibuprofen were selected due to their common usage in treatment of teething symptoms in children [20], and hydrocortisone was selected as a positive control due to its well-known anti-inflammatory effects and mechanisms [21]. Concentrations of the drugs were based on published observations of IC50 values in the inhibition of COX-1 and COX-2: hydrocortisone ~ 5 μM, ibuprofen ~ 8 μM, paracetamol ~ 25 μM [22, 23]. Several concentrations above and below these IC50 values were selected to capture likely effects of each drug on inflammation. After 2 h of incubation with respective drugs or media only, cells were then stimulated by adding LPS (1 μg/mL) from *E. coli* to cause release of prostaglandins and inflammatory cytokines [19]. For quantification of inflammatory cytokines, supernatants were collected after 4 h of LPS stimulation; for quantification of prostaglandins, supernatants were collected after 20 h of LPS stimulation. Cytokines and prostaglandins were then quantified by enzyme-linked immunosorbent assay (ELISA), with three independent experiments performed for each assay.

### Cytokine ELISA

In order to quantify cytokine release by activated THP-1 cells, ELISA was performed for the pro-inflammatory cytokines IL-1α, IL-1β, IL-8 and TNFα using kits from BioLegend (Thermo Fisher Scientific; San Diego, USA). All assays were performed following the manufacturer’s instructions. Briefly, ELISA plates were coated in primary anti-cytokine antibody overnight and washed twice. Culture supernatant samples or cytokine standards were added to the plates and incubated for 3 h on a plate shaker and washed twice. An enzyme-conjugated secondary antibody was then added to the plates and incubated for 2 h on a plate shaker and washed twice. Tetramethylbenzidine (TMB) substrate was then added to plates and left to develop for 15 min before the reaction was stopped by addition of 1 M H_2_SO_4_. Optical density was immediately read using an ELISA plate reader (iMark Microplate Reader, Bio-Rad) at 450 nm.

### Prostaglandin ELISA

In order to quantify prostaglandin release by activated THP-1 cells, ELISA was performed for PGE2 using a kit from Enzo Life Sciences according to the manufacturer’s instructions. Briefly, PGE2 standards and culture supernatants were incubated in wells of a pre-coated plate in the presence of PGE2-enzyme conjugate and anti-PGE2 antibody for 2 h on a plate shaker and washed three times. Paranitrophenylphosphate (pNpp) substrate was then incubated for 45 min before the reaction was stopped by addition of trisodium phosphate stop solution. Optical density was immediately read using an ELISA plate reader at 405 nm.

### Statistical analysis

Standard curves from HLPC analysis to quantify succinic acid were generated by linear regression, and standard curves from ELISA analysis to quantify cytokines were generated by 4-parameter logistic regressions. Each ELISA test was performed three times in independent experiments; for each experiment data were normalised against the LPS-only control, such that these control cells were 100%, thus a value of 200% indicates twice as much as the control and a value of 50% indicates half as much as the control. Normalised ELISA data were analysed by ANOVA using a non-parametric model with Holm-Sidak post-hoc comparisons. A cut-off alpha level of *p* = 0.05 was selected for determining statistical significance, and Prism v7 (Graphpad software, San Diego, CA) was used for all analyses.

## Results

**Fig. 1:**
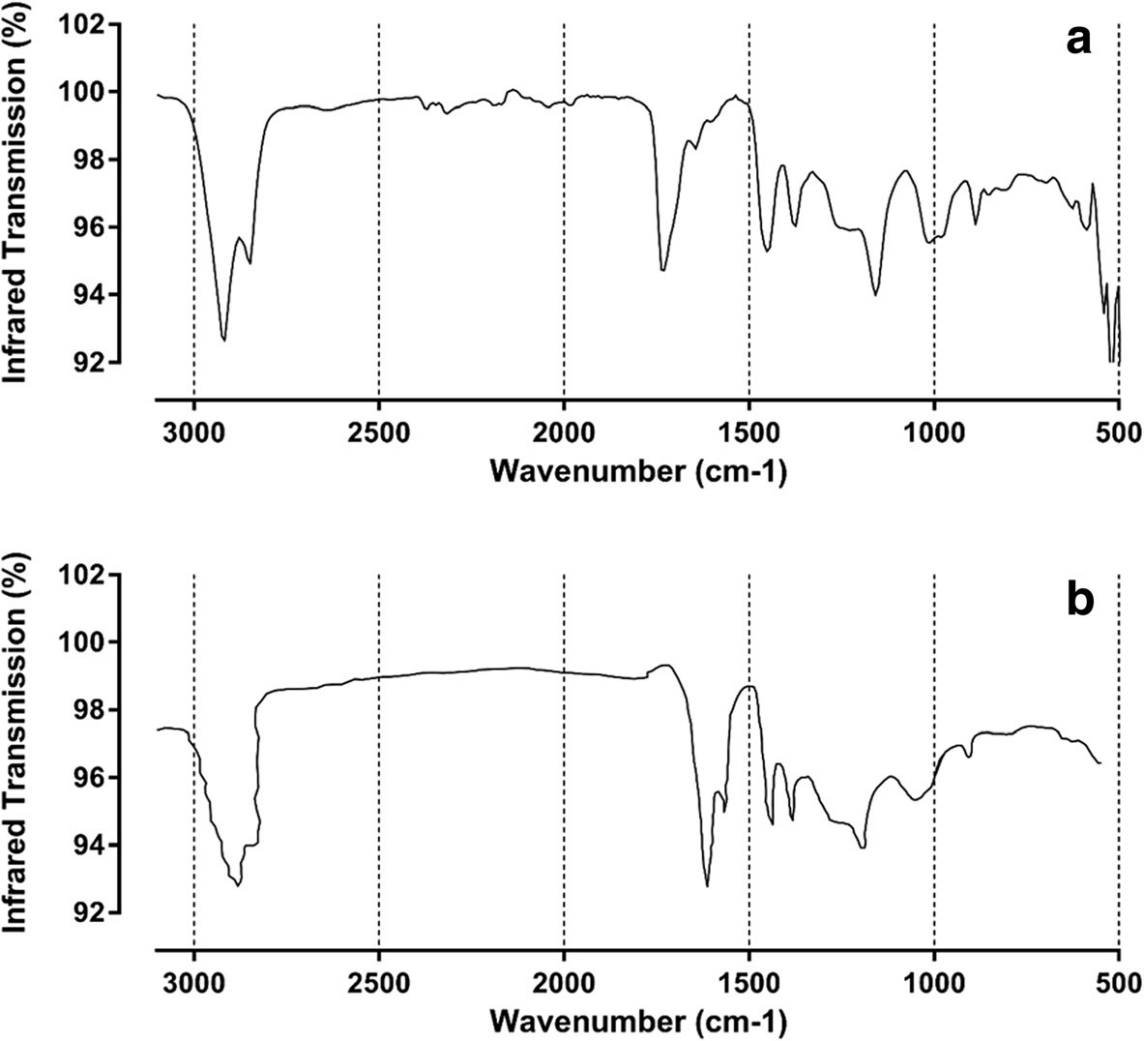
Infrared spectrum of teething necklaces. **a** Crushed amber beads were analysed by infrared spectroscopy, measuring transmittance in the wavenumber range of 500–3100 cm^− 1^. **b** Historical data on the infrared spectrum of Baltic amber was gathered from Beck et al (1956) [17] and adapted using the WebPlotDigitizer v3.21 tool for comparison

Beads weighed 104 ± 24.9 mg (mean ± std. dev of 85 beads weighed individually) with no difference between light, medium and dark bead colours. When powder from finely crushed amber beads were analysed by infrared spectroscopy (Fig. 1), the transmittance mode spectra of crushed teething necklace beads closely resemble that published for Baltic amber [17]. A region called the “Baltic shoulder” from 1160 to 1260 cm^− 1^ is due to the presence of esterified succinic acid and is used to identify Baltic origin amber [8, 24]. In the transmittance mode presented herein, the Baltic shoulder is clearly visible as the horizontal region followed by a trough at around 1200 cm^− 1^ (Fig. 1). Therefore, it was concluded that the purchased amber teething necklaces were made from Baltic amber as advertised.

**Fig. 2:**
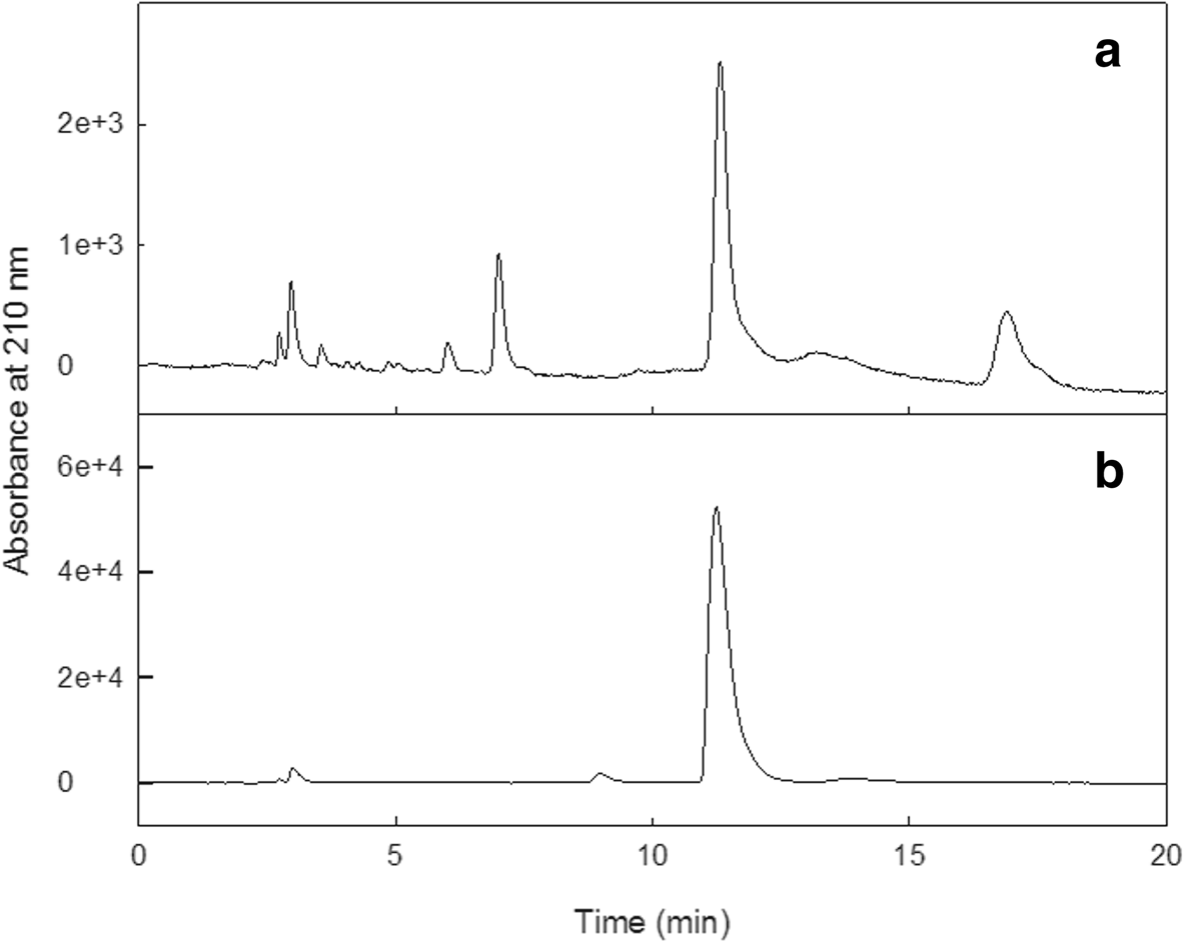
HPLC analysis of amber bead extract and succinic acid. **a** HPLC UV chromatogram (210 nm) from light-coloured amber beads that fragmented when submerged in octanol, showing a peak of succinic acid at 11.2 min. **b** UV chromatogram of pure succinic acid dissolved in octanol as a standard control, showing a peak of succinic acid at 11.2 min

**Fig. 3:**
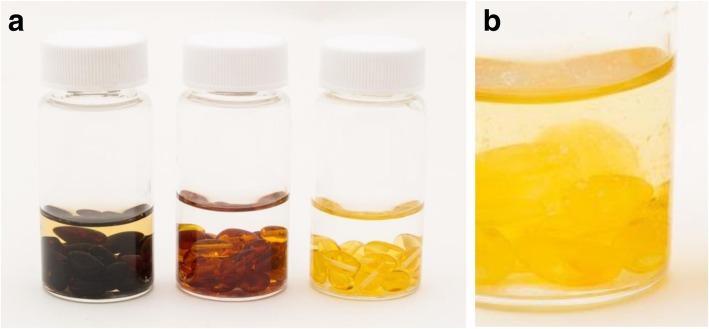
Incubation of amber beads under various biologically relevant conditions. **a** Beads were removed from necklaces and sorted into colour variants, then stored at 37 °C in either pH 5.5 PBS or octanol for 6 months. Bead supernatants were periodically collected and succinic acid content was analysed by HPLC. **b** Succinic acid was only detected in the supernatant of light-coloured beads stored in octanol, in which one of three replicates had significantly fragmented and degraded

HPLC analysis of amber beads dissolved in sulfuric acid (Fig. 2) provided a measurement of 1.5 ± 0.4 mg of succinic acid per bead. As the average bead weight was 104 mg, succinic acid content was 1.44% w/w. For a complete necklace containing 33 beads, this equated to approximately 50 mg of succinic acid. No succinic acid release from beads was detected during submersion in pH 5.5 PBS for 24 weeks. Similarly, no succinic acid release was detected for medium or dark beads submerged in octanol for 24 weeks. In contrast, light-coloured beads that were submerged in octanol fragmented into shards (Fig. 3), and this was associated with a small amount of succinic acid being released (0.73 mg from 22 beads after 4 weeks, no further increase). This is equivalent to 0.033 mg per bead, or 0.03% w/w. The standard curve was linear with an R^2^ of 0.9999, LOD of 0.018 mg/mL and LOQ of 0.055 mg/mL. Inter-day precision was 2.02%. It was concluded that amber beads contain succinic acid, but are unlikely to release succinic acid from whole beads in contexts relevant to the physiological conditions of human skin layers.

**Fig. 4:**
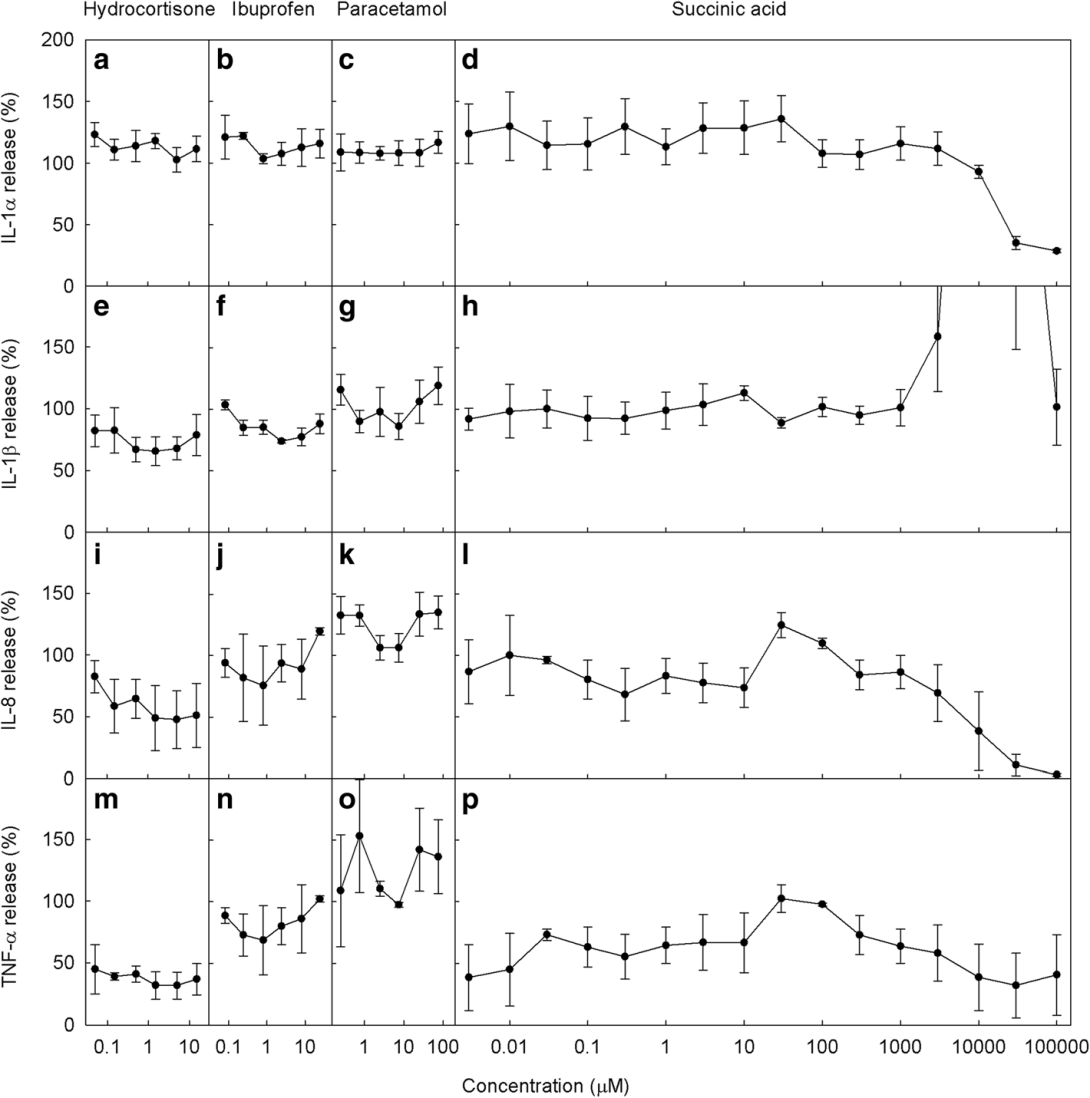
The effects of succinic acid and anti-inflammatory drugs on inflammatory cytokine production. THP-1 human macrophage cells were stimulated with LPS in the presence of the indicated concentrations of each drug and the production of various inflammatory cytokines after 4 h of stimulation were measured by ELISA. Results are presented on a scale that is normalised against results for LPS-only cells. Culture supernatant concentrations of IL-1α (**a**-**d**), IL-1β (**e**-**h**), IL-8 (**i**-**l**) and TNFα (**m**-**p**) in the presence of hydrocortisone (**a**, **e**, **I**, **m**), ibuprofen (**b**, **f**, **j**, **n**), paracetamol (**c**, **g**, **k**, **o**) or succinic acid (**d**, **h**, **l**, **p**). Points represent mean ± SE of 3 independent experiments

**Fig. 5:**
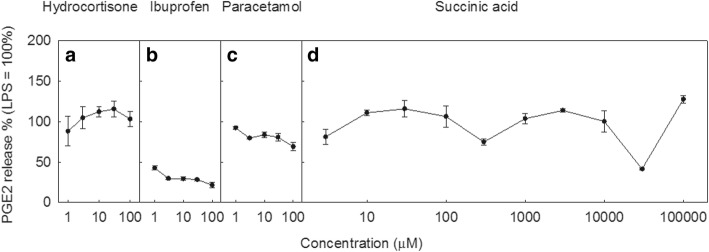
The effects of succinic acid and anti-inflammatory drugs on PGE2 production. THP-1 human macrophage cells were stimulated with LPS in the presence of the indicated concentrations of each drug and the production of PGE2 was measured by ELISA after 20 h of stimulation was measured by ELISA. Results are presented on a scale that is normalised against results for LPS-only cells. Culture supernatant concentrations of PGE2 from macrophages treated with hydrocortisone (**a**), ibuprofen (**b**), paracetamol (**c**) or succinic acid (**d**). Points represent mean ± SE of 3 independent experiments

Ibuprofen and paracetamol had no consistent effects on the release of any studied cytokines **w**hen tested on THP-1 cells stimulated with LPS (Fig. 4). Ibuprofen caused a strong inhibition of PGE2 release, and paracetamol had a weak but significant effect (Fig. 5). Hydrocortisone had no effect on release of IL-1α or PGE2, but significantly decreased the release of IL-1β, IL-8 and TNFα. This strong effect of hydrocortisone was taken to indicate that the 2 h incubation was long enough for the drugs to take effect in these assays; as the mechanism of action of hydrocortisone is via regulation of transcription rather than enzyme inhibition it may be expected to be the slowest onset compared to paracetamol and ibuprofen.

Succinic acid demonstrated no consistent reduction in the release of pro-inflammatory cytokines (Fig. 4) or PGE2 (Fig. 5). At very high concentrations, succinic acid reduced the release of IL-1α and IL-8, and increased the release of IL-1β (604% of LPS-only control at 10 mM). However, the buffer in the culture media was unable to maintain the normal pH range of 6.8 to 7.2 due to the very high concentrations of succinic acid (10–100 mM), resulting in media pH of 3.3 to 3.4 and a distinct yellow-green discolouration. This environment is highly toxic to cells and was most likely causing apoptosis of the THP-1 cells. This cell death would lead to decreased production of cytokines as observed for IL-1α and IL-8 and increased release of IL-1β, which is associated with cell apoptosis via cleavage of pro-IL-1β by the apoptosis-associated enzyme caspase-8. Therefore, it was concluded that succinic acid demonstrated no anti-inflammatory properties at physiologically tolerable concentrations.

## Discussion

Inflammation is a major contributing factor to pain and discomfort associated with teething, as evidenced by the widespread usage of conventional anti-inflammatory or analgesic drugs, such as ibuprofen and paracetamol, in helping to relieve teething pain. Baltic amber teething necklaces have been marketed as an alternative to these pharmacological methods, and putting aside mystical or pseudoscientific claims, the main proposal for a mechanism of action relies on the amber beads being of Baltic origin and containing succinic acid, that the succinic acid can be absorbed from the beads through the skin and this results in anti-inflammatory activity. The current research addresses these claims in a scientific manner for the first time.

We addressed claims of the identity of the amber beads by infrared spectroscopy of crushed beads. Our analysis indicated that the infrared spectrum of crushed beads visually matched published Baltic amber infrared spectra [17, 24]. The ‘Baltic shoulder’ was clearly horizontal, without a slope that could indicate a resin of similar but non-succinite origin [8]. Our findings are limited by the lack of direct comparison with samples of confirmed provenance, or with samples of fake amber, or amber of non-Baltic origin. Furthermore, additional techniques could be applied (e.g. FT-Raman Spectroscopy [25]) to further interrogate the identity of the amber beads in this study. However, FT-IR is a standard and current technique used to identify Baltic amber [24], and based on the investigations that we have conducted, we did not find any reason to dispute the identity of the amber teething necklaces as being genuine Baltic amber.

It is generally considered that Baltic amber contains succinic acid in the range 3–8% w/w [26, 27], but most of this succinic acid is conjugated via ester bonds to the hydroxyl group of other compounds [8]. We measured 1.44% w/w succinic acid by dissolving the macromolecular structure in sulfuric acid, which is expected to have broken the cross-linkages and liberated succinic acid. Extraction in acidified acetonitrile, described as mild conditions and so therefore partial extraction, produced only 0.1% w/w extractable succinic acid from Baltic amber [28], whereas exhaustive extraction of crushed Baltic amber with diethyl ether gave 3.4% succinic acid [8]. As we did not further investigate the presence of succinate complexes in our extract, we may not have obtained a true total succinic acid content for our beads. However, our measurement of significant quantities of succinic acid aligns with our finding that the beads are Baltic amber.

Upon trying to extract succinic acid under conditions relevant to human skin (37 °C, either PBS buffered to pH 5.5 to mimic skin acidity, or octanol to mimic hydrophobic skin conditions), no succinic acid could be extracted from intact beads. A tiny amount of succinic acid was detected only in the supernatant of the light-coloured beads that had spontaneously fragmented into shards. Based on our measurement of 1.44% w/w succinic acid in these amber beads, a 33-bead necklace may be expected to contain approximately 50 mg of succinic acid, and the amount of succinic acid released from these fragmented beads was equivalent to 1 mg per necklace, or 2% of the total succinic acid content of a complete necklace. In Baltic amber only 0.005–0.04% w/w is free succinic acid, determined by extracting powdered Baltic amber with 30% methanol [27]; our measurement is in agreement with this range, equating to 0.03% w/w measured in the fragmented beads. The lack of release of succinic acid from intact beads is a consequence of the majority of it being covalently bonded to other chemicals via ester bonds and playing an integral part of the fossil resin structure [9].

Despite finding no evidence that succinic acid would feasibly be released from amber teething necklaces to be available for absorption via the skin, the anti-inflammatory potential of succinic acid was investigated using LPS-stimulated human macrophages. The inflammatory cytokines IL-1α, IL-1β, IL-8 and TNFα were measured due to their role in pain and inflammation associated with teething and tooth eruption [6]. Treating cells with various concentrations of succinic acid revealed no consistent anti-inflammatory effects until very high concentrations were used. Indeed, at these concentrations of succinic acid, the secretion of IL-1α and IL-8 did decrease. However, in contrast to this, the secretion of IL-1β increased dramatically, up to 6-fold higher concentrations, when treated with 10 mM succinic acid. Succinic acid has been previously documented as increasing IL-1β secretion [29]. Also, given the association of IL-1β secretion with the process of apoptosis [30], and the highly acidic pH of the media in which these cells were cultured, it is likely that cells subjected to these conditions were undergoing apoptotic cell death, which would also account for the decrease in the secretion of IL-1α and IL-8 seen at these concentrations of succinic acid.

While no previous evidence of succinic acid affecting inflammatory cytokine production exists, succinic acid has been shown to reduce the function of human leukocytes, specifically neutrophils. High concentrations of succinic acid (10–30 mM) significantly inhibits motility and phagocytic function of neutrophils, an immune-suppressive effect resulting in increased bacterial infection [31]. This effect of succinic acid also directly contradicts the claims made by several amber necklace retailers that the beads are “immune-stimulating” [2–5].

Of the four drug treatments tested (ibuprofen, paracetamol, hydrocortisone and succinic acid) for their effect on cytokines, only hydrocortisone displayed consistent anti-inflammatory effects, causing a decrease in the secretion of IL-1β, IL-8 and most strikingly TNFα. The inhibitory effects of hydrocortisone on these cytokines have been widely reported [32, 33]. Additionally, previous literature suggests that neither ibuprofen nor paracetamol have consistent inhibitory effects on these specific cytokines [34–40], which is supported by the current research. Analgesia and anti-inflammatory properties of ibuprofen is carried out through other means, namely working as COX inhibitors. Therefore we also investigated the potential of succinic acid as a COX inhibitor by measuring prostaglandin release from macrophages. Ibuprofen displayed strong inhibition of PGE2 synthesis, and paracetamol caused a weak but significant inhibition across the concentration range tested, which is consistent with previous findings regarding these drugs [23]. In our hands hydrocortisone did not inhibit PGE2 release, which is inconsistent with previous research regarding the effects of glucocorticoids on prostaglandin synthesis [22]. However, previous research on this topic has included an overnight pre-incubation step with glucocorticoids rather than the short 2 h pre-incubation used in the current research, which may explain why we saw no effect of hydrocortisone on PGE2 release. Succinic acid did not cause any consistent reduction in PGE2 release, and thereby cannot replicate the effects of other analgesics on either inflammatory cytokines or other common inflammatory molecules.

## Conclusions

To summarise, the current research demonstrated that the purchased amber teething necklaces are genuine Baltic amber, and do contain succinic acid. However, succinic acid could not feasibly be released from intact beads in order to be absorbed through the skin, nor would succinic acid carry out anti-inflammatory effects via the inflammatory mediators tested here even if it was absorbed. The testing undertaken here is not exhaustive, and further study could consider other cell types, such as dendritic cells, which may be more receptive to succinic acid stimulation [41] than the macrophages used herein. Further study could also consider whether other relevant bioactive chemicals could leach out of amber beads and be absorbed through the skin. However, based on these results, we can find no evidence to suggest that the succinic acid component of Baltic amber is likely to provide any anti-inflammatory effects that could be useful in treating teething symptoms.

## Data Availability

The datasets used and/or analysed during the current study are available from the corresponding author on reasonable request.

## Abbreviations

ACCC: Australian Consumer and Competition Commission
COX: Cyclooxygenase
ELISA: Enzyme linked immunosorbent assay
HPLC: High performance liquid chromatography
IL: Interleukin
LOD: Limit of detection
LOQ: Limit of quantitation
LPS: Lipopolysaccharide
NSAID: Non-steroidal anti-inflammatory drug
PBS: Phosphate buffered saline
PGE2: Prostaglandin E2
PMA: Phorbol myristate acetate
pNpp: Paranitrophenylphosphate
PVDF: Polyvinylidene difluoride
TGA: Therapeutic Goods Administration
TMB: 3,3′,5,5′-tetramethylbenzidine
TNF: Tumor necrosis factor

## References

[1] Holistic Baby. Natural ideas, options and diet reccomendations. 2018. https://www.holisticbaby.co.nz/Advice/BABYS+POSITIVE+ADVICE+SOLUTIONS+AND+RESOURCES./Teething/Natural+ideas+options+and+diet+recommendations..html. Accessed 21 Oct 2018.

[2] Teething Made Easy. Amber Info. 2018. http://www.teethingmadeeasy.com/amber-info.html. Accessed 21 Oct 2018.

[3] Amber Artisans. Baltic Amber Alternative Medicine. 2018. https://www.amberartisans.com/baamalme.html. Accessed 21 Oct 2018.

[4] Amber by Amanda. Benefits of amber. 2018. https://amberbyamanda.com/benefits-of-amber-1.html. Accessed 21 Oct 2018.

[5] Snuggle Bugz. Amber teething necklaces - myth or magic?? 2012. http://blog.snugglebugz.ca/amber-teething-necklaces-myth-or-magic/. Accessed 21 Oct 2018.

[6] Shapira J, Berenstein-Ajzman G, Engelhard D, Cahan S, Kalickman I, Barak V (2003). Cytokine levels in gingival crevicular fluid of erupting primary teeth correlated with systemic disturbances accompanying teething. Pediatr Dent.

[7] Cashman JN (1996). The mechanisms of action of NSAIDs in analgesia. Drugs..

[8] Stout EC, Beck CW, Kosmowska-Ceranowicz B, Anderson KB, Crelling JC (1995). Gedanite and gedano-succinite. Amber, Resinite, and fossil resins.

[9] Poulin JH (2014). K. inside amber: the structural role of succinic acid in class Ia and class id resinite. Anal Chem.

[10] Therapeutic Goods Administration. Decision under Regulation 9 of the Therapeutic Goods Regulation 1990 in relation to an advertisement about the products “Amber Necklace, Amber Bracelet, Wonder Balm and Bottom Balm” (Complaint No 2011–09-020). 2013. https://www.tga.gov.au/advert-complaint/amber-necklace-amber-bracelet-wonder-balm-and-bottom-balm-natures-child-pty-limited-complaint-no-2011-09-020. Accessed 21 Oct 2018.

[11] Therapeutic Goods Administration. Decision under Regulation 9 of the Therapeutic Goods Regulation 1990 in relation to an advertisement about the product “Amber Teething Necklace” (Complaint No 2011–09-019). 2013. https://www.tga.gov.au/advert-complaint/amber-teething-necklaces-eco-child-complaint-no-2011-09-019. Accessed 21 Oct 2018.

[12] Australian Competition & Consumer Commission. Consumer protection notice no. 35 of 2011 - safety warning notice (Amber teething necklaces). 2011. https://www.productsafety.gov.au/publication/consumer-protection-notice-no-35-of-2011-safety-warning-notice-amber-teething-necklaces. Accessed 21 Oct 2018.

[13] Queensland Government. Amber teething necklaces. 2017. https://www.qld.gov.au/law/your-rights/consumer-rights-complaints-and-scams/product-safety-for-consumers/safety-advice-and-warnings/baby-products/amber-teething-necklaces. Accessed 21 Oct 2018.

[14] Taillefer A, Casasoprana A, Cascarigny F, Claudet I (2012). Port de colliers de dentition chez le nourrisson. Arch Pédiatrie.

[15] Cox C, Petrie N, Hurley KF (2017). Infant strangulation from an amber teething necklace. CJEM..

[16] Amber by Amanda. FAQ: Help! How do I choose?? 2018. https://amberbyamanda.com/faq-help-how-do-i-choose%2D%2D4.html. Accessed 21 Oct 2018.

[17] Beck C, Wilbur E, Meret S, Kossove D, Kermani K (1965). The infrared spectra of amber and the identification of Baltic amber. Archaeometry..

[18] International Council on Harmonisation - Quality. Validation of Analytical Procedures: Text and Methodology Q2(R1). 2005. https://www.fda.gov/Drugs/GuidanceComplianceRegulatoryInformation/Guidances/ucm065005.htm. Accessed 21 Oct 2018.

[19] Rahiman SSF, Morgan M, Gray P, Shaw PN (2016). Dynorphin 1-17 and its N-terminal biotransformation fragments modulate lipopolysaccharide-stimulated nuclear factor-kappa B nuclear translocation, interleukin-1beta and tumor necrosis factor-alpha in differentiated THP-1 cells. PLoS One.

[20] Tsang AKL (2010). Teething, teething pain and teething remedies. Int Dent South Africa.

[21] Coutinho AE, Chapman KE (2011). The anti-inflammatory and immunosuppressive effects of glucocorticoids, recent developments and mechanistic insights. Mol Cell Endocrin.

[22] Goppelt-Struebe M, Wolter D, Resch K (1989). Glucocorticoids inhibit prostaglandin synthesis not only at the level of phospholipase A2 but also at the level of cyclo-oxygenase/PGE isomerase. Br J Pharmacol.

[23] Cryer B, Feldman M (1998). Cyclooxygenase-1 and cyclooxygenase-2 selectivity of widely used nonsteroidal anti-inflammatory drugs. Am J Med.

[24] Wagner-Wysiecka E (2018). Mid-infrared spectroscopy for characterization of Baltic amber (succinite). Spectrochim Acta A: Mol Biomol Spec.

[25] Edwards HGM, Farwell DW (1996). Fourier-transform Raman spectroscopy of amber. Spectrochim Acta A.

[26] Bogdasarov MA (2007). Mineralogy of fossil resins in northern Eurasia. Geol Ore Deposit.

[27] Tonidandel L, Ragazzi E, Traldi P (2009). Mass spectrometry in the characterization of ambers. II. Free succinic acid in fossil resins of different origin. Rapid Commun Mass Spectrom.

[28] Truica GI, Teodor ED, Litescu SC, Radu GL (2012). LC-MS and FT-IR characterization of amber artefacts. Cent Eur J Chem.

[29] Tannahill GM, Curtis AM, Adamik J, Palsson-McDermott EM, McGettrick AF, Goel G (2013). Succinate is an inflammatory signal that induces IL-1beta through HIF-1alpha. Nature..

[30] Hogquist KA, Nett MA, Unanue ER, Chaplin DD (1991). Interleukin 1 is processed and released during apoptosis. Proc Natl Acad Sci U S A.

[31] Rotstein OD, Pruett TL, Fiegel VD, Nelson RD, Simmons RL (1985). Succinic acid, a metabolic by-product of Bacteroides species, inhibits polymorphonuclear leukocyte function. Infect Immun.

[32] Castro R, Zou J, Secombes CJ, Martin SA (2011). Cortisol modulates the induction of inflammatory gene expression in a rainbow trout macrophage cell line. Fish Shellfish Immunol.

[33] Dovio A, Sartori ML, Masera RG, Peretti L, Perotti L, Angeli A (2004). Effects of physiological concentrations of steroid hormones and interleukin-11 on basal and stimulated production of interleukin-8 by human osteoblast-like cells with different functional profiles. Clin Exp Rheumatol.

[34] Dimova S, Hoet PH, Dinsdale D, Nemery B (2005). Acetaminophen decreases intracellular glutathione levels and modulates cytokine production in human alveolar macrophages and type II pneumocytes in vitro. Int J Biochem Cell Biol.

[35] Endres S, Whitaker RE, Ghorbani R, Meydani SN, Dinarello CA (1996). Oral aspirin and ibuprofen increase cytokine-induced synthesis of IL-1 beta and of tumour necrosis factor-alpha ex vivo. Immunol..

[36] Lacour S, Antonios D, Gautier JC, Pallardy M (2009). Acetaminophen and lipopolysaccharide act in synergy for the production of pro-inflammatory cytokines in murine RAW264.7 macrophages. J Immunotoxicol.

[37] Mascagni P, Sabbatini V, Biordi L, Martinotti S, Allegretti M, Marullo A (2000). R- and S-isomers of nonsteroidal anti-inflammatory drugs differentially regulate cytokine production. Eur Cytokine Netw.

[38] Ribel-Madsen S, Bartels EM, Stockmarr A, Borgwardt A, Cornett C, Danneskiold-Samse B (2012). A synoviocyte model for osteoarthritis and rheumatoid arthritis: response to ibuprofen, betamethasone, and ginger extract - a cross-sectional in vitro study. Arthritis..

[39] Shahriari S, Rezaei A, Jalalzadeh SM, Mani K, Zamani A (2011). Effect of ibuprofen on IL-1beta, TNF-alpha and PGE2 levels in periapical exudates: a double blinded clinical trial. Iran J Immunol.

[40] Syggelos SA, Giannopoulou E, Gouvousis PA, Andonopoulos AP, Aletras AJ, Panagiotopoulos E (2007). In vitro effects of non-steroidal anti-inflammatory drugs on cytokine, prostanoid and matrix metalloproteinase production by interface membranes from loose hip or knee endoprostheses. Osteoarthr Cartil.

[41] Rubic T, Lametschwandtner G, Jost S, Hinteregger S, Kund J, Carballido-Perrig N (2008). Triggering the succinate receptor GPR91 on dendritic cells enhances immunity. Nat Immunol.

